# Alumni Meeting—University of Michigan

**Published:** 1893-12

**Authors:** U. D. Billmeyer


### Alumni Meeting—University of Michigan.

A called meeting of the Alumni of the Dental Department of the University of Michigan was held at Hotel Brunswick, Chicago, August 17, 1893, in the parlors of the Delta Sigma Delta Fraternity.

M. F. Finley, of Washington, D. C., class of '78, was elected President, and U. D. Billmeyer, of Chattanooga, Tenn., class of '80, was elected Secretary.

The following members registered: V. H. Jackson, Marie Thompson Bacon, D. M. Cattell, L. N. Seymour, T. J. Mason, R. M. Paine, T. W. Beckwith, H. F. Harvey, E. D. Brower, M. A. Mason, Geo. H. Wilson, L. L. Barber, Chas. A. Eckert, L. P. Bethel, Wm. Cleland, Helen L. Searle, Vida A. Latham, May Weston, J. W. Wassell, E. Phillips, Geo. J. Dennis, Chas. F. Noyes, P. J. Kesler, John A. Watling, U. D. Billmeyer, J. W. Youngman, C. E. Meerhoff, G. H. Copp, J. C. St. John, Wms. Donnally, W. H. Whistlar, J. Taft, L. L. Davis, M. F. Finley, W. Mitchell, May Cleo Smith, Thomas C. Leiter, Ida Gray.

Very interesting addresses were made by Drs. V. H. Jackson, J. A. Watling, J. Taft, M. F. Finley, Marie T. Bacon, P. J. Kesler and others. After a general handshaking and exchange of greetings, the meeting adjourned to meet at Old Point Comfort next year, at the time of the meeting of the American Dental Association.

U. D. Billmeyer, Secretary.

